# Transcriptomic analysis reveals enhanced host inflammatory responses associated with TNF-α/NF-κB signaling pathway during infection with the ASFV CN/SC/2019 pA104R-K5R

**DOI:** 10.1016/j.virusres.2026.199822

**Published:** 2026-10-25

**Authors:** Xinting Lan, Yikang Chen, Zhonghui Zhang, Jifei Yang, Rongzeng Hao, Xuesai Li, Hualin Sun, Yuting Liao, Zhancheng Tian, Guiquan Guan, Qingli Niu, Hong Yin

**Affiliations:** a State Key Laboratory of Animal Disease Control and Prevention, College of Veterinary Medicine, Lanzhou University, Lanzhou Veterinary Research Institute, Chinese Academy of Agricultural Sciences, Lanzhou, Gansu, 730000, China; b Professional Laboratory for African Swine Fever (Lanzhou), Gansu Province Research Center for Basic Disciplines of Pathogen Biology, Lanzhou, Gansu, 730046, China; c Jiangsu Co-Innovation Center for the Prevention and Control of Important Animal Infectious Disease and Zoonosis, Yangzhou University, Yangzhou, Jiangsu, 225009, China

**Keywords:** African swine fever virus, pA104R, RNA-Seq, Innate immunity, TNF-α/NF-κB signaling

## Abstract

The African swine fever virus (ASFV) is a highly contagious double-stranded DNA virus that belongs to the *Asfarviridae* family. Structurally, ASFV is more complex than other viruses and encodes numerous proteins within each layer. These proteins modulate viral replication in the host via a range of complex molecular mechanisms. Among them, the putative histone-like protein pA104R, which is a late viral protein, plays a crucial role in several stages of the viral life cycle. Our previous study demonstrated that lysine 5 (K5) of pA104R is essential for mediating ubiquitination-induced degradation of pA104R, and that the K5R mutant virus caused severe host inflammation in vivo. However, the underlying mechanism remains unclear. In this study, we examined the mechanism by which the pA104R mutant strain enhances ASFV replication in the host at the transcriptome level. Compared with the WT group, the mutant strain group induced more severe host inflammation by increased expression of inflammatory mediators associated with the TNF-α/NF-κB pathway and the NLRP3 inflammasome, as shown by transcriptome sequencing and Western blot. The results also suggested that ASFV CN/SC/2019 pA104R-K5R induced distinct transcriptional changes in multiple innate immune-related genes. Additionally, the ELISA results demonstrated a higher level of inflammation in cells infected with the mutant strain. This evidence suggested its potential to enhance inflammatory pathology, though further in vivo studies are needed to confirm this. This study provides new insights into the functions of key structural proteins of ASFV in viral replication and host immune modulation. It also offers important theoretical support for elucidating ASFV pathogenic mechanisms.

## Highlights

• Transcriptomic analysis reveals a distinct and stronger host response to the ASFV CN/SC/2019 pA104R-K5R mutant virus compared with the wild-type virus.
• The K5R mutant virus induces extensive host transcriptional alterations, characterized by the significant upregulation of genes associated with inflammatory and immune responses.
• The enhanced inflammatory phenotype of the K5R strain is associated with increased TNF-α/NF-κB inflammatory signaling and the NLRP3 inflammasome.

## 1 Introduction

African swine fever (ASF) is a severe haemorrhagic viral disease caused by African swine fever virus (ASFV), which poses a major threat to the global pig industry, with a case fatality reaching up to 100% (Dixon et al., 2020; Galindo and Alonso, 2017; Revilla et al., 2018). In addition to domestic pigs and wild boars, ASFV can also infect soft ticks of the *Ornithodoros.* spp. ASFV can be persistently transmitted between domestic and sylvatic environments through soft ticks, and the virus can survive for up to eight years in ticks (Ekue and Wilkinson, 1990; Wilkinson et al., 1977). Once ASFV is established in ticks, long-term viral maintenance, evolution, and transmission may lead to the formation of natural endemic foci. This tick-mediated persistence of the life cycle substantially complicates ASFV eradication and presents an obstacle to effective disease control.

ASFV is the sole member of the *Asfarviridae* family. It possesses a large and complex double-stranded DNA genome ranging from approximately 170 to 190 kb that encodes 150–200 viral proteins (Rodríguez and Salas, 2013). These proteins play essential roles in viral replication, assembly, survival, and host interaction (Alejo et al., 2018). During virion maturation, the major core shell polyproteins pp220 and pp62 are cleaved into several mature products by the viral protease pS273R. The process is critical for the formation of infectious ASFV particles (Rodríguez et al., 2006). In addition, several non-structural proteins have been shown to participate in viral replication and assembly. For example, pA151R is thought to be involved in ASFV replication and virion assembly. In summary, all these studies highlight the functional diversity and importance of ASFV proteins (Huang et al., 2020).

In addition to the above functions, ASFV proteins also modulate host signaling pathways, either suppressing or activating host immune responses, thereby influencing viral virulence and pathogenesis. These pathways include key targets such as innate immune signaling and inflammatory responses. This was demonstrated in several studies on the functions of ASFV proteins in these pathways. The conserved ASFV protein I329L has been reported to interact with host TRIF, thereby inhibiting TLR3-mediated NF-κB activation and IFN-β induction (Correia et al., 2013; de Oliveira et al., 2011).

The histone-like protein pA104R is present primarily in the nucleoid of mature ASFV particles and functions as a DNA-associated protein. It plays a critical role in viral DNA replication, repair, recombination, and transcription (Simões et al., 2015). pA104R can bind to both double-stranded and single-stranded viral DNA. It also interacts with ASFV-encoded topoisomerase II to promote DNA supercoiling, which is vital for efficient viral genome replication (Coelho et al., 2015, 2016; Freitas et al., 2016; García-Beato et al., 1992). Most research has primarily focused on the DNA-binding properties of pA104R. Our previous study has demonstrated that the host factor RNF2 suppresses ASFV replication by mediating K48-linked polyubiquitination of pA104R at lysine 5 (K5), thereby targeting it for proteasomal degradation (Zhang et al., 2026). Another study shows that pA104R becomes highly enriched in the matrix attachment regions of viral factories during the late stage of infection (Frouco et al., 2017). These studies suggest that the functions of pA104R are not limited to viral genome replication and also play an important role in the study of virus-host interactions.

Although several ASFV proteins have been implicated in regulating host immune responses, the contribution of pA104R to virus–host interactions-particularly in the modulation of host inflammation-remains largely unexplored. In this study, we investigated the role of pA104R during ASFV infection by comparing the transcriptomic profiles of porcine alveolar macrophages (PAMs) infected with the ASFV CN/SC/2019 pA104R-K5R mutant strain versus the parental ASFV CN/SC/2019 strain. Combining transcriptomic analysis with pathway enrichment and molecular validation approaches, we examined how the pA104R mutant virus affects host inflammatory signaling networks, with a particular focus on TNF/NF-κB-associated pathways and inflammasome-related responses. These analyzes provide a framework for understanding the involvement of pA104R in host immune regulation and offer new insights into its potential role in ASFV pathogenesis.

## 2 Materials and methods

### 2.1 Biosafety and ethics statement

All experiments involving live ASFV were performed in the Biosafety Level 3 (BSL-3) Laboratory of the Lanzhou Veterinary Research Institute (LVRI), Chinese Academy of Agricultural Sciences (CAAS). This laboratory was accredited by the China National Accreditation Service for Conformity Assessment (CNAS) and approved by the Ministry of Agriculture and Rural Affairs of the People's Republic of China. All animal experiments in this study were conducted in accordance with the animal ethics procedures and guidelines of LVRI and CAAS (Approval No.: LVRIAEC-2025–095). To mitigate potential risks, all procedures were conducted under approved BSL-3 protocols, all activities must be monitored by LVRI professionals, and random inspections could be conducted by local and central government authorities without prior notice.

### 2.2 Cell and viruses

Primary porcine alveolar macrophages (PAMs) were isolated from the lung lavage fluid of 30-day-old pigs according to the established bronchoalveolar lavage techniques, and then cultivated in RPMI 1640 medium (Servicebio, G4535–500ML, China) supplemented with 10% fetal bovine serum (FBS; SORFA, SX3000, China) and 1% penicillin–streptomycin solution (Solarbio, P7630, China) at a humidified 37 °C and 5% CO2 atmosphere. The ASFV CN/SC/2019 strain was isolated, identified, and maintained in the BSL-3 laboratory of the LVRI, CAAS. The ASFV CN/SC/2019 pA104R-K5R with enhanced green fluorescent protein (eGFP) (Zhang et al., 2026) was generated from the parental strain ASFV CN/SC/2019 and maintained in LVRI-BSL-3.

### 2.3 Antibodies

Polyclonal antibodies (1:500) against the p72, p30, and pA104R proteins were stored in our laboratory. The following antibodies were procured: anti-β-actin mouse monoclonal antibody (1:20,000, Proteintech, 66,009–1), anti-NLRP3 rabbit antibody (1:1000, CST, 15101S), anti-IL-1β rabbit antibody (1:1000, CST, 12703S), anti-Cleaved-IL-1β rabbit antibody (1:1000, CST, 83186S), anti-caspase-1 rabbit antibody (1:1000, CST, 3866S), anti-ASC/TMS1 rabbit antibody (1:1000, CST, 13833S), anti-NF-κB p65 mouse antibody (1:1000, CST, 6956S), anti-phospho-NF-κB pp65 rabbit antibody (1:1000, CST, 3033S), anti-phospho-IkappaB alpha rabbit monoclonal antibody (1:1000, CST, 2859S), anti-IkappaB alpha antibody (1:1000, CST, 9242S), anti-AIM2 rabbit monoclonal antibody (1:1000, CST, 12948S), Goat anti-Mouse IgG (H+L) Secondary Antibody (1:10,000, Invitrogen, 31,430), Goat anti-Rabbit IgG (H+L) Secondary Antibody (1:10,000, Invitrogen, 31,460).

### 2.4 RNA extraction and transcriptome sequencing

PAMs were prepared in 12-well plates. Infections were performed within the designated time frame using ASFV CN/SC/2019 or ASFV CN/SC/2019 pA104R-K5R at a multiplicity of infection (MOI) of 1.0, followed by total RNA extraction using the Trizol reagent (Invitrogen, 15,596,026, USA). RNA quality was assessed on an Agilent 2100 Bioanalyzer (Agilent Technologies, Palo Alto, CA, USA) and checked using RNase free agarose gel electrophoresis. After total RNA was extracted, polyadenylated mRNA was selectively captured using Oligo (dT) primers (Thermo, SO131, USA).

The mRNA was fragmented into 200-bp fragments and reverse transcribed into cDNA using the NEB Next Magnesium RNA Fragmentation Module (NEB, E6150S, USA). First-strand cDNA was synthesized using random hexamers, followed by second-strand cDNA synthesis. End repair was performed by adding A-Tailing Mix and RNA Index Adapters. The cDNA fragments were amplified by PCR and purified using Ampure XP beads. The resulting cDNA library was sequenced using Illumina Novaseq 6000 by Gene Denovo Biotechnology Co. (Guangzhou, China). The differential expression of mRNAs was analyzed using DESeq2 software, v. 3.20 with thresholds set at |log₂FC| > 1 and FDR < 0.05. Transcripts that did not meet both criteria were not classified as DEGs.

### 2.5 Functional enrichment of DEGs analysis and protein interaction network analysis

The differential expression genes were subjected to Gene Ontology (GO) enrichment and Genes and Genomes (KEGG)analysis using data from the GO database (http://www.geneontology.org (accessed on 20 December 2024)), KEGG database (https://www.genome.jp/kegg/pathway.html (accessed on 20 December 2024)) and Reactome database (https://reactome.org (accessed on 20 December 2024)), respectively. protein interaction networks were analyzed by using the STRING database (https://cn.string-db.org/ (accessed on 20 December 2024)).

### 2.6 Real-time quantitative PCR (RT-qPCR)

To validate the reliability of RNA-Seq high-throughput sequencing results, selected up-regulated and down-regulated genes were subjected to RT-qPCR analysis. PAMs were infected by ASFV CN/SC/2019 or ASFV CN/SC/2019 pA104R-K5R for 24 h. Total RNA was extracted from each sample using Trizol (Invitrogen, 15,596,026, USA), and cDNA was synthesized using the RT Master Mix for qPCR II (gDNA digester plus) (MCE, HY-K0511A, USA) according to the manufacturer’s instructions. Quantitative real-time PCR assays were performed to calculate the relative mRNA expression levels of the genes via the 2^^-ΔΔCT^ method, with GAPDH as the internal reference gene for normalization. The primer sequences are listed in Table 1. The data were analyzed using GraphPad Prism 10.2.1 software.

**Table 1:** The primers used in this study.

GenBank Number	Primers	Sequences
NM_001267892.1	MPEG1-F	tcaggctgccaccaacactg
	MPEG1-R	gcagcacctggaaccgaact
NM_001256366.1	MARCO-F	tagcggtgtctgggggacaa
	MARCO-R	gcctgcgtcctcattgtggt
NM_213,942.1	C4BPA-F	tgcctccttggggtcctgtt
	C4BPA-R	tgcaggtgaccagcgtgaag
XM_021070736.1	PROS1-F	cctagcgcttcccgtcttgg
	PROS1-R	gacagccgggcagcagtaaa
NM_214,209	ACP5-F	ctgccactgctgaccacaca
	ACP5-R	ttgcgcaggtgcttcttgga
NM_001033015	ACE-F	tcaccaacaaggctgccagg
	ACE-R	cagcagatgggctgggatgg
NM_001114281.1	RELA-F	tgcttccaggtgacagtgcg
	RELA-R	cccgagttccgattcacccg
NM_214,055	IL-1β-F	agggacatggagaagcgattt
	IL-1β-R	ttctgcttgagaggtgctgatg
NM_214,022.1	TNF-α-F	ggctgccttggttcagatgt
	TNF-α-R	caggtgggagcaacctacagtt
NM_001256775.2	CCL14-F	cctcctcctcctcatcgcca
	CCL14-R	ctctcgggattgcacgggtc
XM_021072741.1	NFKB2-F	atggaggagctggggttggt
	NFKB2-R	taccagaggttgggggcgaa
NM_001256770	NLRP3-F	tgcactgactggcagaaggc
	NLRP3-R	ccacatgccgaggatggtcc
NM_214,029	IL-1α-F	gattcgcaacttcctgtgac
	IL-1α-R	tgatggttttgggtgtctca
NM_214,041.1	IL10-F	tacctggaagacgtaatgcc
	IL10-R	aactcacccatggctttgta
NM_213,867	CXCL8-F	aagcttgtcaatggaaaagag
	CXCL8-R	ctgttgttgttgcttctcag
NM_001198921.1	ISG12-F	tcttatcctctagcctggct
	ISG12-R	gctcctagtgaagaagccat
Q29599.3	OAS1-F	catccctagagggccatgag
	OAS1-R	aaaggagacagagcacggaa
396,655	STAT1-F	gtgggaccgcaccttcagcc
	STAT1-R	agggccagcagtgggaccaa
NM_002046	GAPDH-F	gattccacccatggaaattc
	GAPDH-R	ctggaagatggtggtgatgggatt

### 2.7 ELISA assay

Following infection of PAMs with ASFV CN/SC/2019 and ASFV CN/SC/2019 pA104R-K5R at 24 h, the supernatant was collected and then stored at −80 °C for analysis. The cell supernatant was used to measure the concentrations of TNF-α (PTA00, R&D Systems) and IL-1β (P8000, R&D Systems). Each sample was assayed in triplicate in strict accordance with the manufacturer’s protocols. The absorbance was read at a wavelength of 450 nm using an ELISA reader, and the concentrations of inflammatory cytokines were calculated using the standard curve method. All of the data were analyzed using GraphPad Prism software (version 10.2.1, GraphPad), and the results are reported as the means ± standard deviations.

### 2.8 Western blot analysis

Cell samples were collected and lysed in RIPA buffer supplemented with a protease and phosphatase inhibitor cocktail (Thermo Scientific, Cat No 78,442), followed by incubation on ice for 2 h. The mixture was subsequently centrifuged at 12,000 rpm for 10 min at 4 °C to remove unlysed cell debris, after which we transferred the supernatant to a new tube. Protein concentrations were evaluated using the BCA protein assay reagent (Thermo Scientific, Cat No 23,225), and the samples were stored at −80 °C for subsequent Western blot analysis. Protein samples were denatured in a water bath at 100 °C for 10 min after adding loading buffer. Equal amounts of total protein (20 μg per lane) were separated by SDS- PAGE, and then transferred to nitrocellulose (NC) membranes (Merck-Millipore, HATF00010, USA). The membranes were blocked with 5% (w/v) skimmed milk for 2 h at room temperature, then incubated with the specified primary antibody overnight at 4 °C. Following washes with PBST, the membrane was incubated with the corresponding secondary antibody for 1 h at room temperature. Protein bands were treated with enhanced chemiluminescence (ECL) reagent (Thermo Fisher Scientific, 32,106, USA) and detected using the Crescendo ECL detection system (BIO-RAD, 1705,061, USA) via chemiluminescence and autoradiography.

### 2.9 Quantification and statistical analysis

Statistical analyzes were conducted with Prism 10.2.1 software (GraphPad, San Diego, CA). The significance of differences between the experimental and control groups was analyzed by one-way analysis of variance (ANOVA). Two-tailed *p* values were calculated, and *p* values < 0.05 were considered to indicate statistical significance (*p < 0.05, **p < 0.01,***p < 0.001 and ****p < 0.0001). ns: Non-significant difference.

## 3 Results

### 3.1 Effects of the pA104R K5R mutation on viral protein expression and sample-level transcriptomic profiles

To investigate the effect of ASFV CN/SC/2019 pA104R-K5R on viral protein expression and host transcription in vitro*,* porcine alveolar macrophages (PAMs) were seeded in 12-well plates and infected with ASFV CN/SC/2019 (WT group) or ASFV CN/SC/2019 pA104R-K5R (K5R group) at a multiplicity of infection (MOI) of 1.0 for 12, 24, and 48 h. Uninfected PAMs were used as the negative control group (NC group). Cell samples were collected at different time points for immunoblotting, and samples at 24 h were collected for transcriptome sequencing. Compared with ASFV CN/SC/2019 infection, ASFV CN/SC/2019 pA104R-K5R infection resulted in a significant increase in viral p30 level at 48 hpi. For pA104R, the K5R group exhibited higher protein levels than the WT group at 12, 24 and 48 hpi. Both p30 and pA104R levels increased in a time-dependent manner from 12 to 48 hpi (Fig. 1A, B). The quality control of the sequencing data confirmed that the proportion of Q20 base quality values exceeded approximately 98%, and Q30 values were at least 94% in all samples. Furthermore, GC content distributions were consistent and showed a similar distribution pattern in all samples. These results suggest that the filtered sequencing data were of high quality for subsequent analysis (Table 2). Principal component analysis (PCA) and Pearson correlation coefficient (PCC) analysis were further used to evaluate sample relationships and reproducibility (Fig. 1C). The results showed that samples clustered well within each group and also exhibited similar composition and strong correlations in all samples. Meanwhile, a clear separation was observed between different experimental groups (Fig. 1D). The violin plot showed similar gene expression distributions in all samples, ensuring the same amount of information collected from different transcriptomes (Fig. 1E). It indicated good reproducibility within groups and significant differences in transcription between them.

**Fig. 1:**
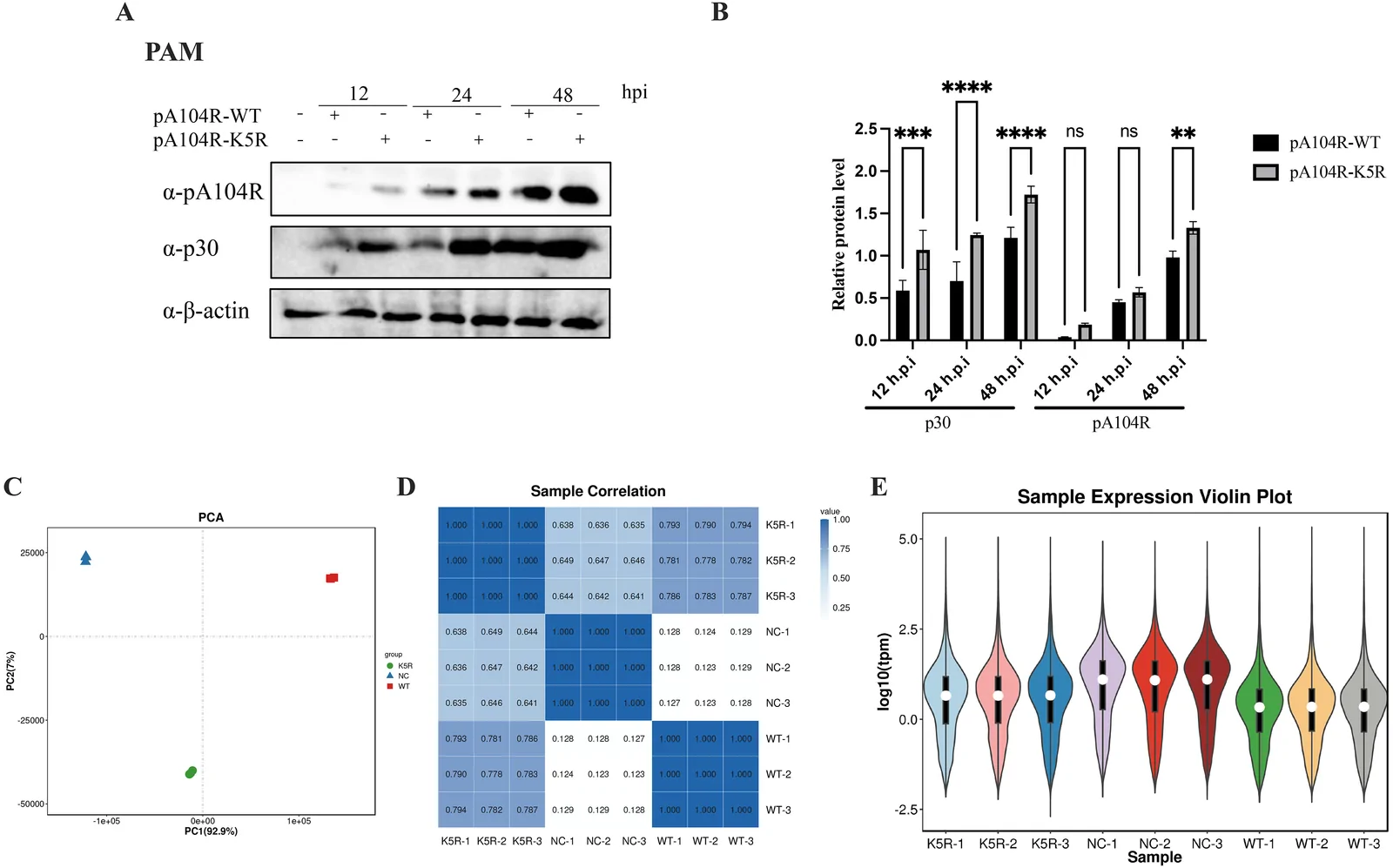
Effects of the pA104R K5R mutation on viral protein expression and sample-level transcriptomic profiles. (A) PAMs were infected or uninfected with ASFV CN/SC/2019 or ASFV CN/SC/2019 pA104R-K5R at an MOI of 1.0 for 12, 24, 48 hpi, followed by Western blot analysis of pA104R, p30 and β-actin expression levels with anti-pA104R, anti-p30, and anti-β-actin antibodies. (B) Relative quantification of p30 and pA104R protein levels from the Western blots in (A). Band intensities were normalized to β-actin, and pA104R-K5R (gray bars) was compared with pA104R-WT (black bars) at the indicated time points. Quantitative data are presented as mean ± standard deviation (SD). Statistical analysis was performed using Student’s *t*-test (n = 3). ** *p* < 0.01, *** *p* < 0.01,**** *p* < 0.0001; ns, no significant difference. (C) Principal component analysis (PCA) analysis. (D) Sample-to-sample distance analysis. The correlation between samples was assessed by listing the identified genes and their respective quantities. A deeper color indicates a high correlation with minimal distance between the samples, while a lighter color reflects a low correlation with greater distance. (E) The Violin plot of sample expression distribution. Each color represents one sample, and the enlarged part represents the region with the most concentrated gene expression.

**Table 2:** Summary of raw sequencing data quality.

Sample	RawData	GC(%)	Q20(%)	Q30(30%)
NC-1	6061,348,500	49.08	98.23	94.86
NC-2	7757,122,200	48.97	98.04	94.38
NC-3	7757,122,200	48.96	97.96	94.09
WT-1	6338,704,800	42.74	98.16	94.52
WT-2	5720,318,700	42.61	98.14	94.45
WT-3	6242,044,800	42.73	98.08	94.36
K5R-1	6402,603,300	47.59	98.17	94.74
K5R-2	6220,041,900	47.62	98.13	94.58
K5R-3	6729,053,700	47.60	98.11	94.63

### 3.2 Transcriptomic profiling reveals an asymmetric DEG landscape and enhanced host inflammatory and immune responses associated with pA104R-K5R

To characterize gene expression patterns among different experimental groups, we first quantified the total number of differentially expressed genes (DEGs) using the DEGs criteria (|log₂ fold-change (FC)| > 1 and FDR < 0.05). Compared with the NC group, 283 genes were up-regulated, and 10,481 genes were down-regulated in the WT group. In contrast, there were 877 up-regulated genes and 7441 down-regulated genes in the K5R group. Notably, comparing the K5R group with the WT group, 8193 genes were up-regulated, while only 50 genes were down-regulated (Fig. 2A). To interpret the highly asymmetrical DEG pattern in the K5R and WT comparison, we performed a Venn diagram. Notably, 7128 of the 8193 genes up-regulated in K5R vs WT (87%) were down-regulated in WT vs NC (Fig. 2B). To further examine the distribution and expression features of these DEGs, we performed a clustered heatmap analysis to visualize overall transcriptional differences. Clear distinctions in gene expression profiles were observed between WT and NC groups, as well as between K5R and NC groups. In the NC-vs-WT group, the up-regulated genes were mainly viral genes and made up 60%, including X69R, E146L, H108R, B119L, and B117L. In contrast, in the NC-vs-K5R group, viral genes accounted for only 19% of the up-regulated DEGs, including X69R, ASFV_G_ACD_00600, E146L and ASFV_G_ACD_00320 (Tables S1, S2). While comparing with the WT group, the genes showing increased expression were predominantly host inflammatory and immune-related factors, such as CCL17, CXCL8, IL-1β, TNF, CCL14, and NLRP3. However, the expressions of PROS1, ACP5, MPEG1, and MARCO were reduced in the K5R group. These down-regulated DEGs were associated with anti-inflammation and innate immune defense (Fig. 2C). Taken together, these results suggested that ASFV CN/SC/2019 pA104R-K5R may contribute to enhancing host inflammatory and immune responses.

**Fig. 2:**
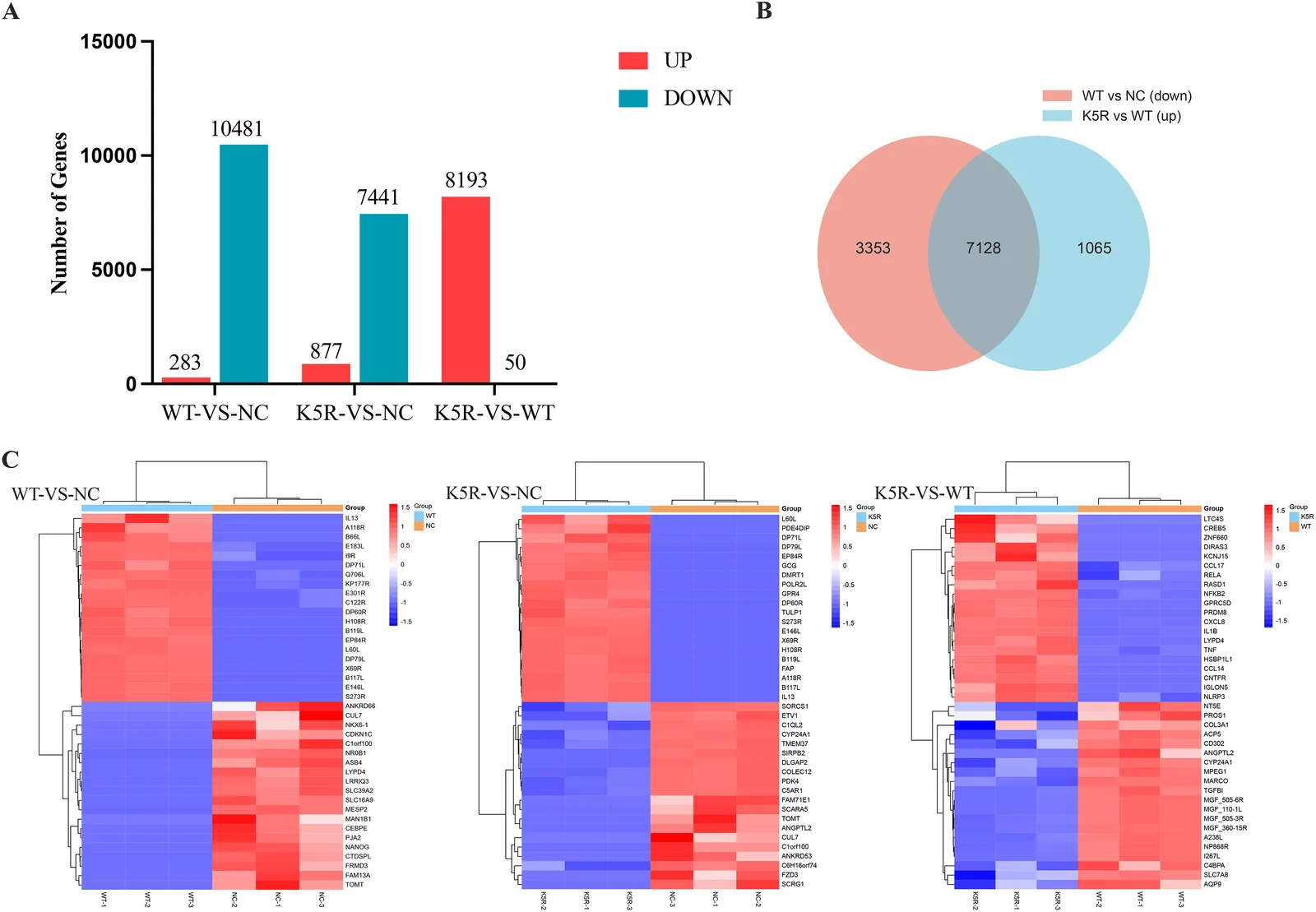
DEG identification and expression profiling in ASFV CN/SC/2019 WT- or pA104R-K5R-infected PAMs. (A) The total number of differentially expressed genes (DEGs) identified in the comparison between NC, WT and K5R groups. (B) The Venn diagram of the down- regulated DEGs in WT vs NC and up- regulated DEGs in K5R vs WT. (C) Heatmap clustering of the top 40 DEGs among the NC, WT, and K5R groups. Each column represents a sample, and each row represents a gene. Red and blue colors indicate higher and lower expression levels (log₂ CPM), respectively.

### 3.3 GO and KEGG enrichment analysis of DEGs reveals metabolic, autophagic, and TNF/NF-κB-associated inflammatory pathway alterations

To explore the potential biological functions of these differentially expressed genes (DEGs) in the host, we performed Gene Ontology (GO) and Kyoto Encyclopedia of Genes and Genomes (KEGG) pathway enrichment analyzes using the top 20 GO terms and pathways ranked by the lowest Q values. For GO annotation, the genes were categorized into three primary ontologies: molecular function (MF), cellular component (CC), and biological process (BP). When comparing the WT and NC groups, GO analysis showed that DEGs were mainly enriched in cellular component (CC) categories, mainly including intracellular and intracellular part (Fig. 3A). The results were the same in the K5R-vs-NC group and K5R-vs-WT group (Fig. 3B, C). These results indicate that the proteins encoded by these genes were primarily localized within the cell and in membrane-enclosed organelles. To investigate the potential roles of the DEGs in immunity and inflammation, the Bar Gradients of BP terms related to immunity and inflammation were constructed. When comparing the WT and NC groups, DEGs were enriched in immune system process, regulation of immune system process and positive regulation of immune system process. In the K5R-vs-NC group, immune system process, immune response and regulation of immune system process were the enriched terms. In the K5R-vs-WT group, DEGs were enriched in response to cytokine, cellular response to cytokine stimulus and leukocyte activation (Fig. S1).

**Fig. 3:**
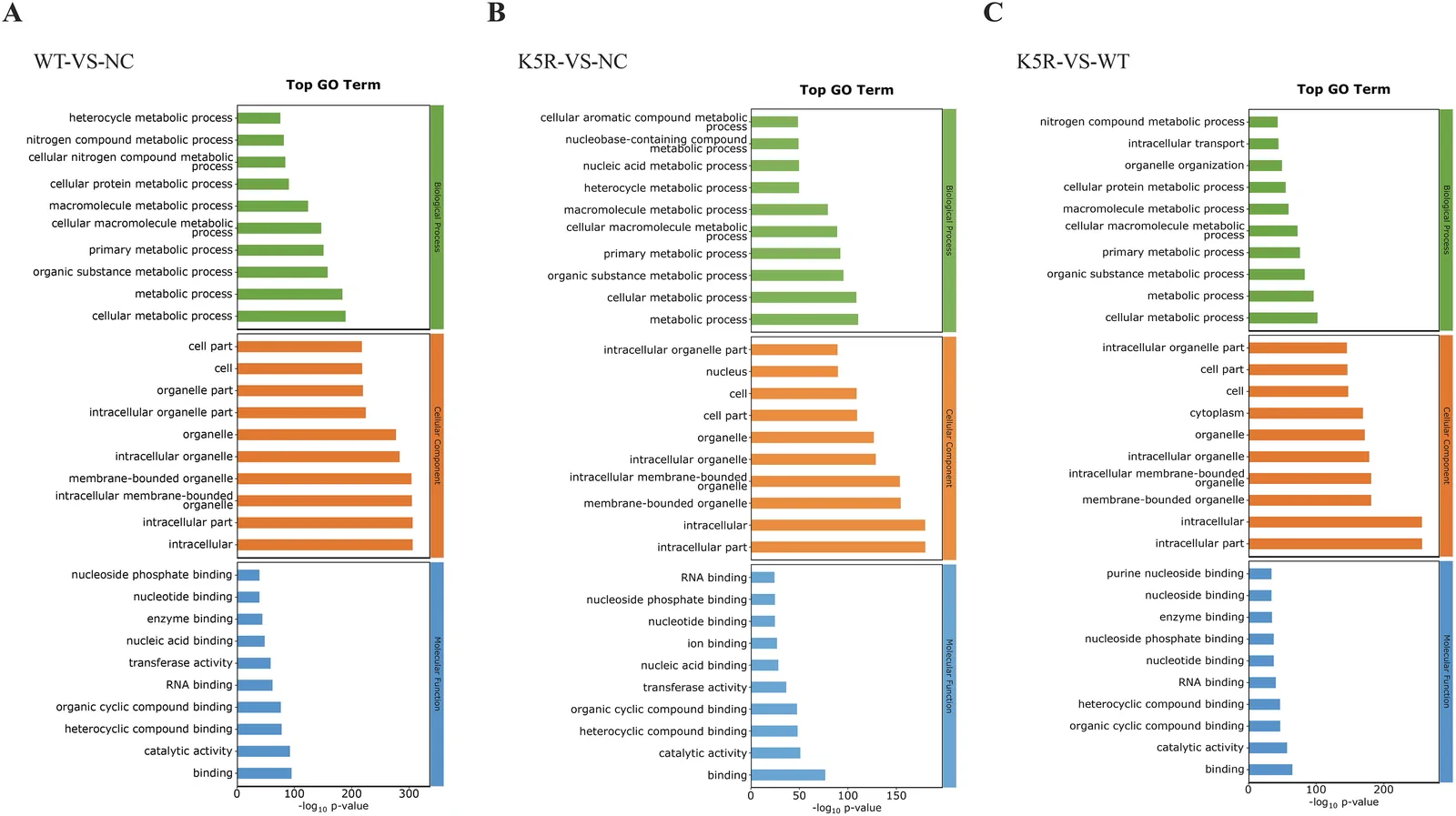
GO enrichment analysis of DEGs in PAMs infected with ASFV CN/SC/2019 WT or pA104R-K5R and in uninfected PAMs based on RNA-Seq data. (A) The Gene Ontology (GO) enrichment analysis of DEGs for WT vs. NC at 24 hpi. (B) GO enrichment analysis of DEGs for K5R vs. NC at 24 hpi. (C) GO enrichment analysis of DEGs for K5R vs. WT at 24 hpi.

KEGG pathway enrichment analysis revealed that, compared with the NC, DEGs in the WT and K5R groups were mainly enriched in Herpes simplex virus 1 infection, Metabolic pathways, Autophagy-animal, Lysosome, and NOD-like receptor signaling pathway. WT and K5R virus infection primarily affected host metabolism, autophagy, and immune responses. Notably, when comparing the K5R group with the WT group, DEGs were significantly enriched in protein processing in endoplasmic reticulum, metabolic pathways, and the TNF signaling pathway (ko04668). 115 genes were identified in the TNF signaling pathway, including 80 up-regulated genes. Compared with the NC group, DEGs in the K5R group were also enriched in the TNF signaling pathway. However, there were only 14 up-regulated and 61 down-regulated genes in this pathway (Fig. 4A, B). This suggested that the mutant virus infection is associated with significant transcriptional alterations in the TNF signaling pathway. Heatmaps were used to analyze the DEGs involved in the TNF signaling pathway and the NF-κB signaling pathway. Several DEGs, including IL-1β, TNF-α, CCL2, TLR4, and RELA were involved in both the TNF signaling pathway and the NF-κB signaling pathway (Fig. 4C). These two major inflammatory pathways suggested that the mutant virus plays an important role in regulating inflammatory responses, cell apoptosis, and innate immune pathways. Taken together, these results suggested that the mutant virus modulated host metabolism and inflammatory responses through complex regulatory mechanisms, which may contribute to the altered host response induced by the mutant strain.

**Fig. 4:**
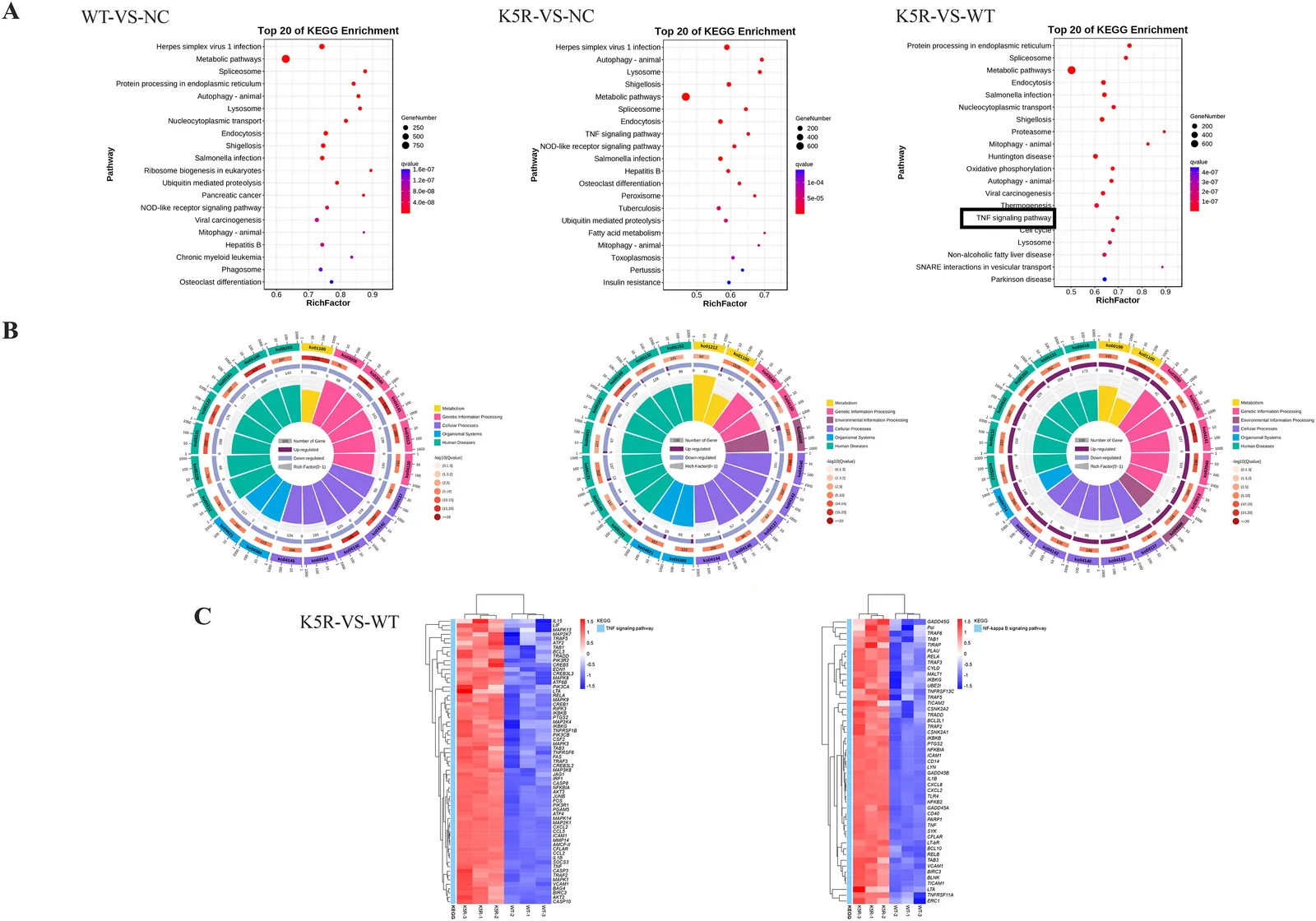
KEGG enrichment analysis of DEGs in PAMs infected with ASFV CN/SC/2019 WT or pA104R-K5R and in uninfected PAMs based on RNA-Seq data. (A) The Kyoto Encyclopedia of Genes and Genomes (KEGG) pathway enrichment analysis for WT vs. NC, K5R vs. NC, and K5R vs. WT at 24 hpi. (B) KEGG enrichment circle plots for WT vs. NC, K5R vs. NC, and K5R vs. WT at 24 hpi. (C) Heatmap of DEGs involved in the TNF and NF-κB signaling pathways (K5R vs. WT at 24 hpi).

### 3.4 Protein-protein interactions (PPI) network analysis of DEGs

To further investigate the biological relevance of the differentially expressed genes (DEGs), we used the STRING database to construct a protein–protein interaction (PPI) network. Compared with the NC group, PPI network analysis of the WT group showed that MMP9, TLR2, and TLR4 were central nodes (Fig. 5A). In the K5R group, proteins such as CCL5, CXCL2, and TLR4 were identified as important hub nodes (Fig. 5B). When comparing the WT and K5R groups, we found that a higher proportion of DEGs in the K5R group occupied central positions in the interaction network. These DEGs, including IL-1β, TNF-α, NLRP3, CCL8, MPEG1, and MARCO, showed extensive interactions within the network (Fig. 5C). The extensive interactions were observed among these proteins, indicating a tightly connected network structure. Functional classification showed that these DEGs were primarily involved in inflammatory responses, innate immune pathways, and pathogen recognition processes.

**Fig. 5:**
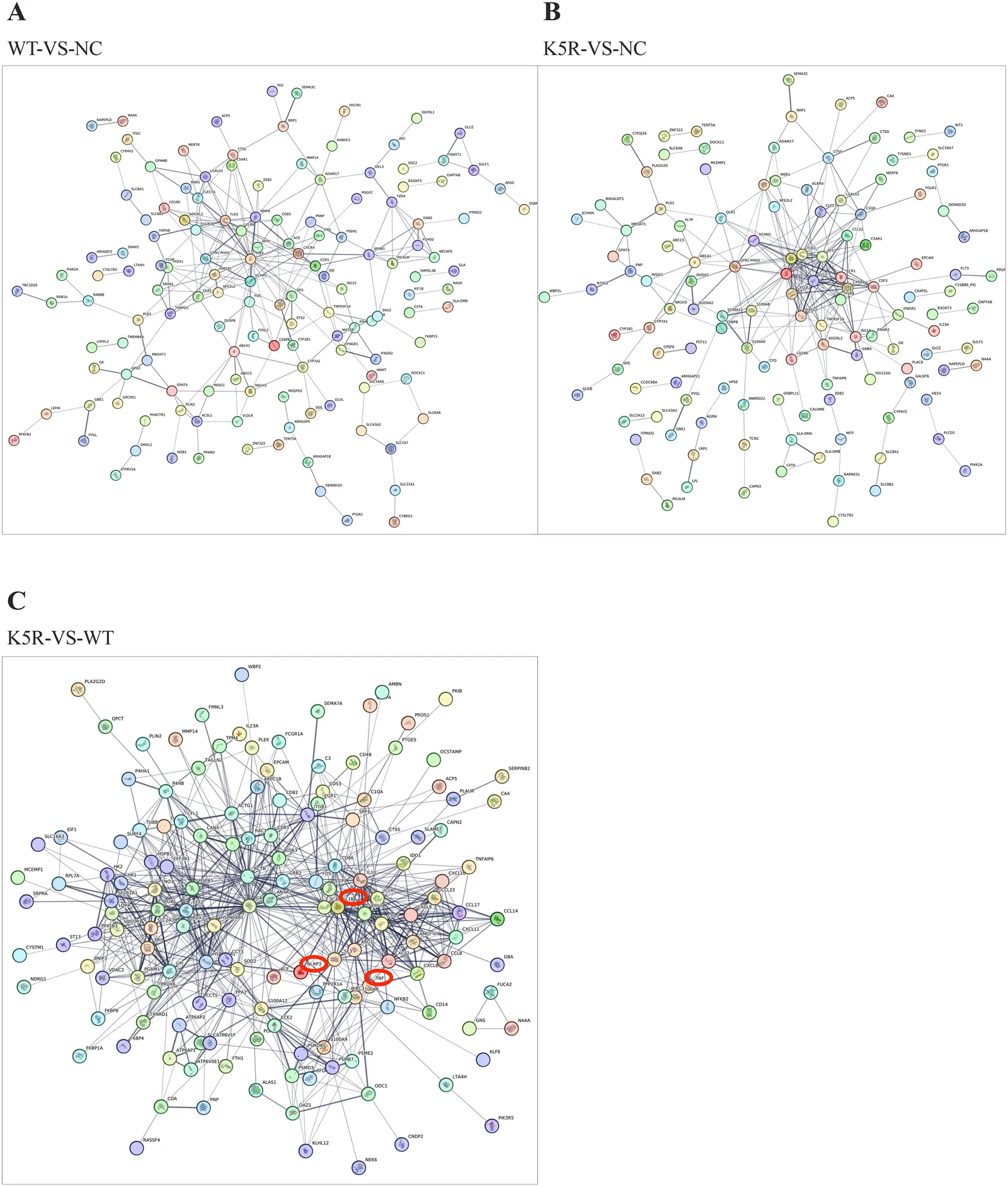
PPI network of DEGs reveals inflammatory and immune-related hub genes in the pA104R-K5R group. The PPI network was constructed using the STRING database to visualize interactions among DEGs defined as |log₂FC| > 1 and FDR < 0.05. The network highlights key hub nodes, including NLRP3, IL-1β, MARCO, TLR4, and TNF, which are implicated in inflammatory responses, immune activation, and pathogen recognition, underscoring their potential roles in mediating ASFV CN/SC/2019 pA104R-K5R effects on host cell processes.

### 3.5 RT-qPCR validation of RNA-Seq data reveals inflammatory gene expression changes in the pA104R-K5R group

To confirm the reliability of the RNA-Seq data, six up-regulated and six down-regulated genes were randomly selected. We used the RT-qPCR method to examine transcription levels of these genes. It was shown that the selected genes displayed clear up- or down-regulation patterns. Importantly, the expression trends of these genes were consistent with those observed in the transcriptomic analysis. This indicates that the RNA-Seq data were reliable and suitable for subsequent analysis. Compared with the WT group, the K5R group exhibited increased transcription levels of RELA, IL-1β, TNF-α, CCL14, NFKB2 and NLRP3, and showed decreased expression of MPEG1, MARCO, C4BPA, PROS1, ACP5 and ACE, especially the high expression of TNF-α and NLRP3 (Fig. 6). Based on the previous results of DEGs and KEGG enrichment analysis (Figs. 2, 4), these transcriptional changes suggest that the mutant virus is associated with enhanced TNF-α/NF-κB inflammatory signaling, leading to a significant alteration of the host transcriptional status.

**Fig. 6:**
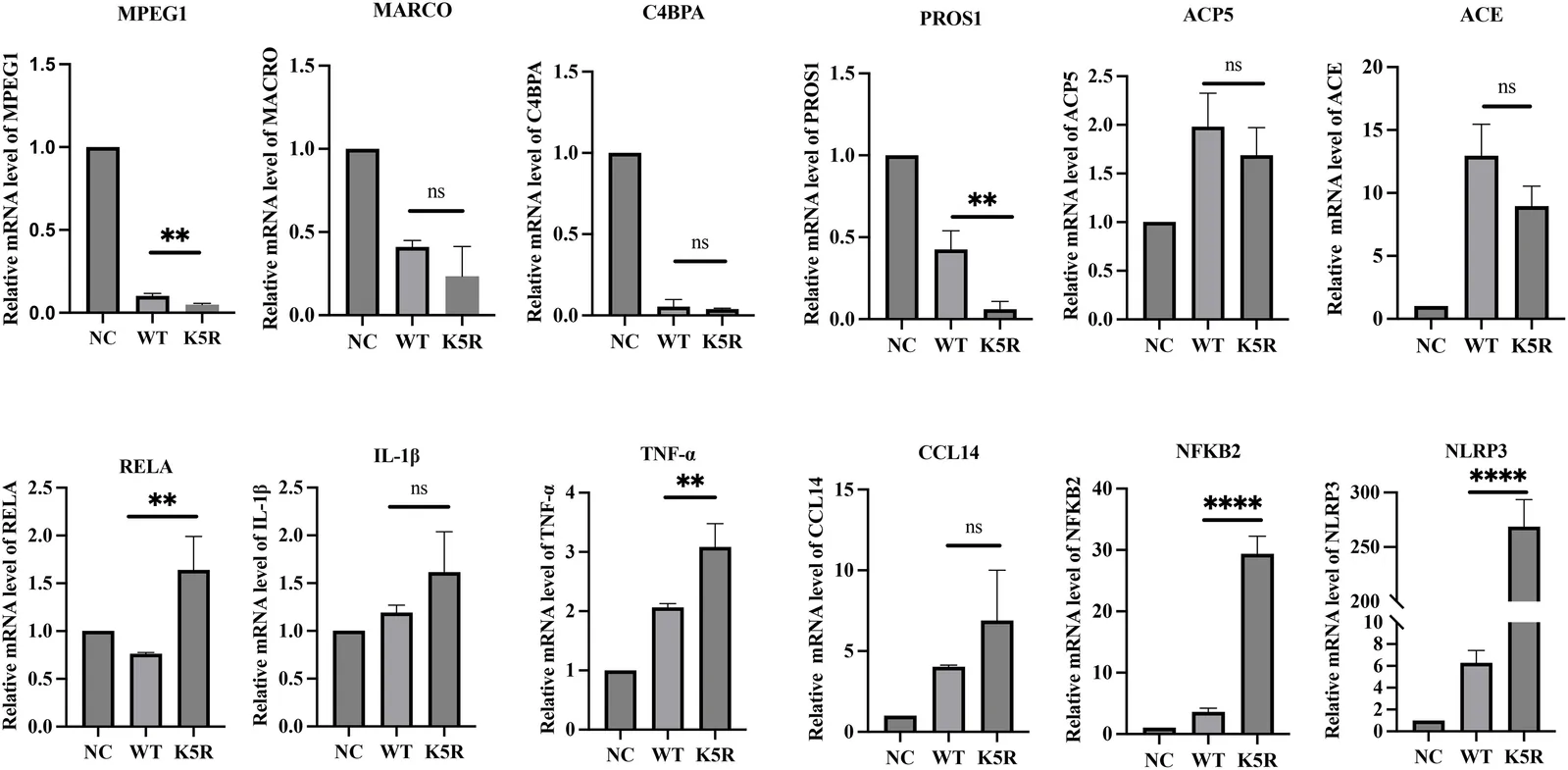
RT-qPCR validation of RNA-Seq data for selected host genes. PAMs were uninfected or infected with ASFV CN/SC/2019 or ASFV CN/SC/2019 pA104R-K5R at 1.0 MOI for 24 hpi. RNA was extracted and RT-qPCR was carried out to test their expression level. Quantitative data are presented as mean ± standard deviation (SD). Statistical analysis was performed using Student’s *t*-test (n = 3). ** *p* < 0.01, **** *p* < 0.0001; ns, no significant difference.

### 3.6 ASFV CN/SC/2019 pA104R-K5R differentially regulates innate immune-related gene expression

To investigate the effect of the ASFV CN/SC/2019 pA104R-K5R on the innate immune response, heatmaps were used to analyze the DEGs involved in the innate immune pathway. Several DEGs, including up-regulated STAT1, NLRP3, IL-1α, IL-10, CXCL8, IL-1β, ISG12 and OAS1 were involved in the innate immune pathway (Fig. 7A). The RT-qPCR method was used to examine the transcription levels of these genes. Compared with the WT group, the K5R group exhibited increased transcription levels of IL-1α, IL-10, and CXCL8, and showed decreased expression of STAT1 and OAS1 (Fig. 7B). The inconsistent trends of STAT1 and OAS1 between RNA-seq and RT-qPCR may reflect methodological differences in transcript isoform detection and quantification. The results suggested that the K5R mutant strain may differentially regulate innate immune genes at the transcriptional level.

**Fig. 7:**
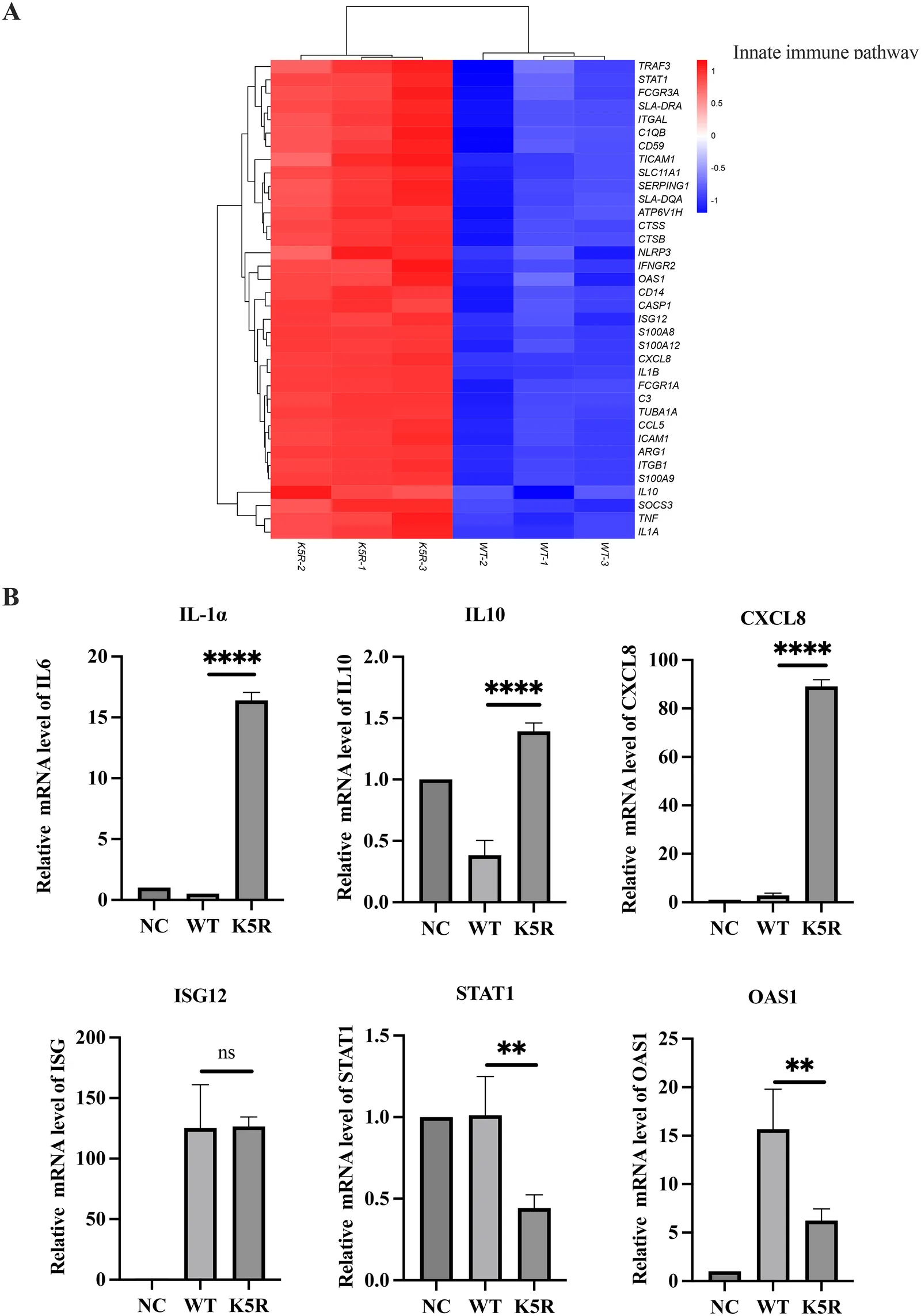
pA104R-K5R modulates the expression of innate immune-related genes. (A) Heatmap of DEGs involved in the innate immune pathway. K5R vs. WT at 24 hpi. (B) PAMs were uninfected or infected with ASFV CN/SC/2019 or ASFV CN/SC/2019 pA104R-K5R at 1.0 MOI for 24 hpi. RNA was extracted and RT-qPCR was carried out to test their expression level. Quantitative data are presented as mean ± standard deviation (SD). Statistical analysis was performed using Student’s *t*-test (n = 3). ** *p* < 0.01, **** *p* < 0.0001; ns, no significant difference.

### 3.7 ASFV CN/SC/2019 pA104R-K5R enhances TNF-α/NF-κB-associated inflammatory signaling and promotes inflammatory responses

Inflammatory responses generally proceed in two sequential steps: priming and triggering. Priming is the preparatory step of the inflammatory responses by upregulating the inflammatory molecules (NF-κB, AP-1), and triggering is the activation stage of the inflammasome (Yi, 2022). To determine whether the mutant virus affects TNF-α/NF-κB–mediated inflammatory signaling, we compared the changes in key NF-κB pathway proteins during infection with the mutant and wild-type strains. The results showed that the protein levels of phosphorylated IκBα (p-IκBα) and phosphorylated p65 (p-p65) were higher in the K5R group than in the WT group. It indicated that NF-κB signaling was further activated upon infection with the mutant virus (Fig. 8A). To assess whether the mutant strain induces an excessive inflammatory response in the host, we analyzed the protein expression of inflammatory mediators as well as cytokine levels in cell supernatant. The expression levels of NLRP3, IL-1β, and ASC increased in the K5R group, consistent with the transcriptional data (Fig. 8B). In addition, ELISA results also confirmed elevated levels of inflammatory cytokines (Fig. 8C). Together, these findings indicate that ASFV CN/SC/2019 pA104R-K5R enhanced TNF-α/NF-κB–associated inflammatory signaling. It was suggested that ASFV CN/SC/2019 pA104R-K5R may contribute to the host inflammatory response.

**Fig. 8:**
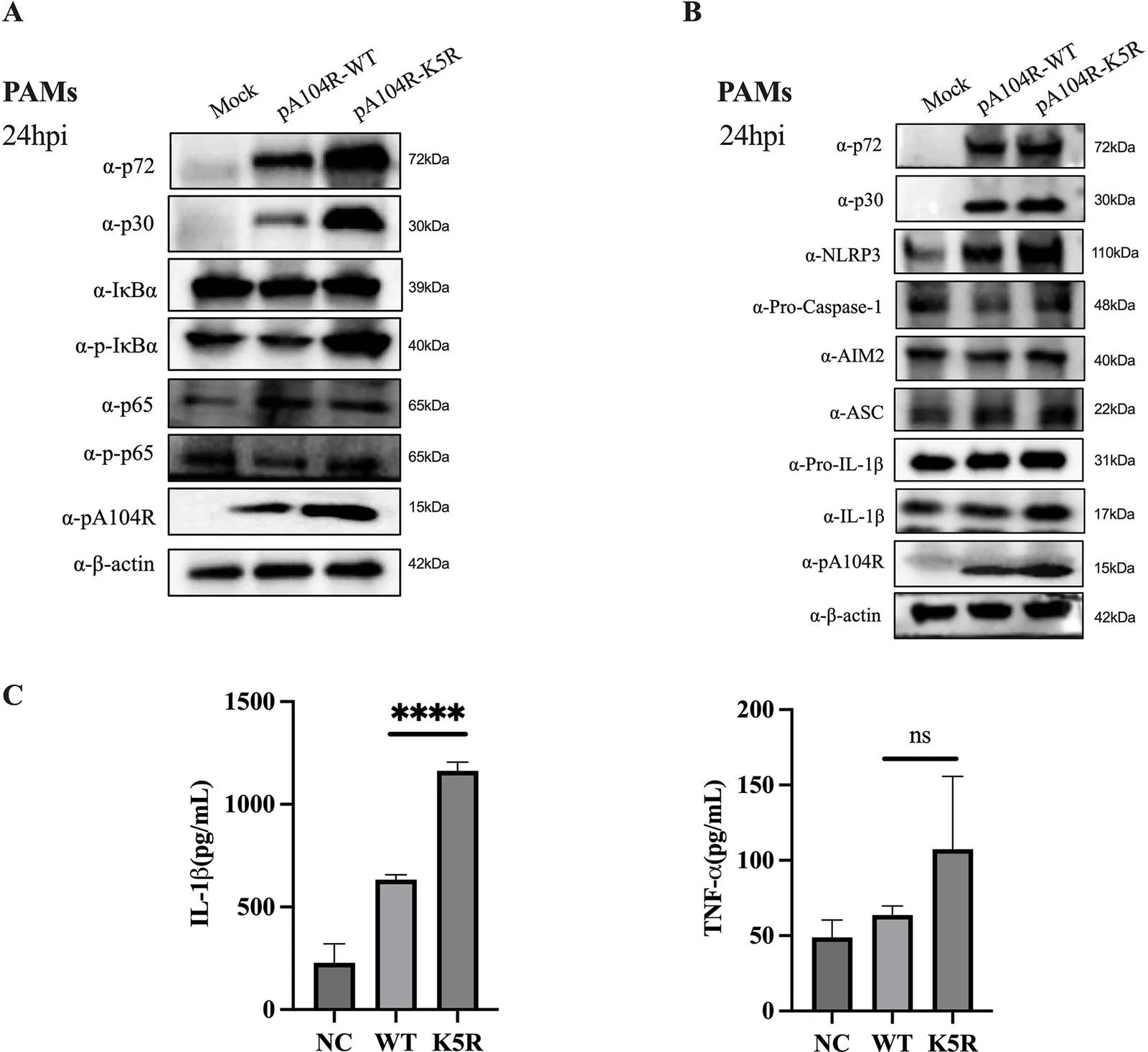
ASFV CN/SC/2019 pA104R-K5R enhances TNF-α/NF-κB signaling and inflammatory responses. (A) PAMs were left uninfected or infected with ASFV CN/SC/2019 or ASFV CN/SC/2019 pA104R-K5R at an MOI of 1.0 for 24 hpi, followed by analysis of protein expression levels related to the NF-κB signaling pathway. (B) PAMs were left uninfected or infected with ASFV CN/SC/2019 or ASFV CN/SC/2019 pA104R-K5R at an MOI of 1.0 for 24 hpi to assess the protein expression levels of inflammatory factors. (C) The levels of IL-1β and TNF-α concentrations in the cell culture supernatants were measured. Quantitative data are presented as mean ± standard deviation (SD). Statistical analysis was performed using Student’s *t*-test (n = 3). **** *p* < 0.0001; ns, no significant difference.

## 4 Discussion

ASF is a highly lethal viral disease that poses a serious threat to the global swine industry. ASFV has a large and complex genome that encodes numerous viral proteins (Solikhah et al., 2025). However, due to limited basic research, the functions of many of these proteins are still poorly explored. Therefore, there is an urgent need to use modern virology, immunology, and bioinformatics approaches to better understand ASFV proteins and clarify their roles in viral replication, pathogenicity, and host signaling regulation.

In recent years, transcriptome analysis has been increasingly used in ASFV research. Increasing evidence suggests that ASFV proteins can change the host transcriptional environment and modulate key signaling pathways, which are crucial for viral replication. For example, transcriptomic analysis has shown that MGF360–2 L plays an important role in viral immune regulation and is associated with enhanced interferon-related transcriptional signatures in infected cells (Zhang et al., 2025). The ASFV protein C315R promotes viral replication by up-regulating IL-6 expression and activating STAT3 phosphorylation via the IL-6/STAT3 signaling axis (Geng et al., 2025). In addition, RNA-seq analyzes with host, parental and deleted virus strains at different times of infection of porcine alveolar macrophages (PAMs) also reveal that I329L could affect the NF-κB pathway by regulating host or viral gene expression (Duan et al., 2023). In summary, these studies underscore the value of transcriptomic analyzes for uncovering the functions of ASFV-encoded proteins and their involvement in host–virus interactions.

The pA104R protein is a highly conserved ASFV protein present in all ASFV isolates (Urbano et al., 2024), suggesting a fundamental role in the viral life cycle. Despite its conservation, the influence of pA104R on the host transcriptional programs has not been systematically investigated. In this study, we used transcriptome analysis to compare the host transcriptional responses and viral gene expression profiles after infection with wild-type and mutant viruses. Our data showed that the pA104R function is associated with significant changes in host transcriptional profiles. Compared with the WT group, the expression of several inflammatory and immune-related genes has increased in the K5R group, including IL-1β, TNF, NLRP3, CXCL8, and CCL17. The pathway enrichment analysis also revealed significant changes in metabolic pathways as well as in TNF and NF-κB signaling pathways. These results suggest that the mutant virus is associated with enhanced inflammatory and immune signaling. Thereby, it ultimately influences host responses during infection. Our study thus provides new transcriptomic evidence for the role of pA104R in regulating host responses during ASFV infection.

Accumulating evidence has demonstrated that ASFV proteins are closely associated with host inflammatory regulation. The inflammatory response is a central part of innate immunity, and several ASFV proteins have been reported to affect this process. For example, the I177L protein induces host inflammatory responses by promoting the TRAF6–TAK1 axis and NLRP3 inflammasome assembly (Wu et al., 2025). In contrast, ASFV strains deficient in CD2v and MGF360/505 suppress NF-κB signaling and reduce IL-1β production (Gao et al., 2021). Moreover, an iGLuc reporter (pro-IL-1β-Gluc) of pig origin studies have identified several ASFV-encoded proteins, including S183L, E199L, O61R, and I7L. They are activators of inflammatory responses (Song et al., 2020). The pA104R is the only histone-like protein encoded by eukaryotic viruses and is involved in viral transcription, DNA replication, and genome packaging (Frouco et al., 2017). This underscores its importance in maintaining efficient viral propagation. In addition to its structural and replicative functions, the pA104R sequence contains a histone-like protein feature. The sequence plays a critical role in promoting stable, organized, compact nucleoid and heterochromatinization of the host cell genome (Borca et al., 1996; Salas and Andrés, 2013). The pA104R has also been shown to activate host innate immunity by suppressing IFN-α–induced ISG expression and ISRE promoter activation. It suggests that it contributes to immune evasion during ASFV infection (Chen et al., 2023a). Consistently, deletion of the A104R drastically reduces virus virulence in domestic pigs (Ramirez-Medina et al., 2022). All this evidence indicates that pA104R may play multiple roles in coordinating viral replication, immune modulation, and pathogenicity.

Based on these studies, our findings provide new insight into how disruption of pA104R may alter host immune responses and inflammatory responses. Transcriptomic analyzes revealed that infection with the pA104R mutant virus was associated with enhanced expression of a broader set of inflammatory and innate immune–related genes compared with WT virus infection. Notably, several important mediators of inflammatory signaling, including IL-1β, TNF, NLRP3, and several chemokines, were consistently up-regulated. These transcriptional changes were the same as the increased protein expression of representative inflammatory mediators and inflammasome components. This suggested that alteration of pA104R at the K5 site is associated with enhanced inflammation in infected cells. Such an exaggerated inflammatory response may influence the local immune microenvironment.

Both WT and K5R infection induced significant changes in the host transcriptional profile, and the extent of these changes differed markedly between the two viruses. Compared with the NC group, there were 283 upregulated and 10,481 downregulated genes in the WT group, whereas 877 upregulated and 7441 downregulated genes were detected in the K5R group. The large number of downregulated genes following ASFV CN/SC/2019 infection suggests more extensive suppression of host transcription by this strain. This number exceeds that reported in previous studies, where only a few thousand downregulated DEGs were detected at 24 hpi (Cao et al., 2023). This discrepancy may reflect differences in MOI, virus strain, time point, sequencing depth, and DEG calling thresholds. In our study, PAMs were infected at an MOI of 1.0, higher than in several previous studies, which may have contributed to the stronger host transcriptional inhibition observed.

Further comparison between the K5R and WT groups identified 8193 genes that were upregulated in the K5R group, while only 50 genes were downregulated. However, the K5R mutation partially alters this transcriptional response. The Venn diagram revealed that 7128 (87.0%) of the 8193 genes upregulated in K5R vs WT were downregulated in WT vs NC. The remaining 1065 genes, which were uniquely upregulated in K5R vs WT, may represent genes specifically activated by the K5R mutant. Together with the enhanced ASFV replication observed for the K5R mutant, these findings suggested that the K5R mutation may change the host transcriptional response, resulting in the relative upregulation of a large proportion of genes that are suppressed during ASFV CN/SC/2019 infection.

Compared with the WT group, the K5R group increased the transcriptional levels of IL-1α, IL-10, and CXCL8, which was consistent with the transcriptomic data, suggesting that the K5R virus may alter cytokine-associated immune responses. However, RT-qPCR showed that STAT1 and OAS1 were downregulated. The expression of the two genes was inconsistent with the RNA-seq analysis. This discrepancy may be attributable to biological variation among samples. Previous studies have shown that pA104R antagonizes type I interferon signaling by attenuating STAT1 phosphorylation and suppressing ISRE activity (Chen et al., 2023a). Thus, the K5R mutation may alter the ability of pA104R to regulate host innate immunity. However, the present findings do not support uniform activation of the type I interferon pathway. The K5R mutation may differentially regulate inflammation-related genes and interferon-stimulated genes.

Pathway enrichment analysis further supports these results. The differentially expressed genes of the mutant virus were primarily enriched in the TNF signaling pathway and the NF-κB signaling pathway. These two signaling pathways are central regulators of host inflammatory and immune responses. The inflammatory response in ASFV infection involves numerous immune cells and signaling pathways, notably NF-κB. These responses and pathways are crucial for transcriptional regulation (Casanova and Abel, 2021; Wang et al., 2020; Zuo et al., 2024). NF-κB is a primary transcription factor that modulates the expression of numerous cytokines, chemokines, and cell death–related genes. These functions are essential for maintaining immune homeostasis. Increasing research suggests that ASFV regulates NF-κB signaling through various viral proteins with different functions. For example, it has been reported that the ASFV protein I177L promotes NF-κB activation and the formation of NLRP3 inflammasome, thereby enhancing inflammatory responses (Wu et al., 2025). However, the protein I10L inhibits NF-κB signaling by targeting IKKβ (Chen et al., 2023b). In this study, our protein–protein interaction network analysis showed that IL-1β, TNF, and NLRP3 represent central hub nodes, further implicating these genes in the host response to the pA104R mutant virus. Consistent with these findings, infection with the mutant virus increased transcription of genes of NF-κB signaling pathway, including TNF-α, IL-1β, and RelA, and phosphorylation of the NF-κB subunit p65. In addition, expressions of various inflammasome-associated proteins, such as IL-1β, NLRP3, ASC, and AIM2, were also increased by western blot analysis. Collectively, these findings suggest that pA104R may contribute to TNF/NF-κB–mediated inflammatory signaling during ASFV infection. Disruption of this regulatory balance may lead to excessive inflammatory responses, which have important implications for host immune regulation, cell death pathways, and ASFV-host interactions in PAMs in *vitro*.

In summary, we combined transcriptome profiling with transcriptional and protein-level validation analysis to investigate the host transcriptional and inflammatory response to the pA104R mutant virus in PAMs in *vitro*. This study demonstrates that infection with the mutant virus is associated with enhanced host inflammatory responses and activation of TNF-α/NF-κB–related signaling. Our findings further indicate that the K5R site of pA104R is a critical determinant in host–virus interactions and in promoting ASFV replication.

## 5 Conclusion

In conclusion, this study reveals a key role of ASFV pA104R in the regulation of host inflammatory responses and innate immunity through integrated transcriptomic, molecular, and protein analyzes. Compared with the wild-type virus, the pA104R mutant virus enhances TNF/NF-κB pathway activity in PAMs, which is associated with increased inflammatory cytokine expression and activation of NLRP3 inflammasome–associated molecules. Meanwhile, changes in STAT1, OAS1, and other immune-related genes suggest that the K5R mutant virus may influence the innate immune responses. These findings demonstrate that the K5R site of pA104R is a critical factor influencing host–virus interactions and promoting ASFV replication. Given that pA104R has been previously shown to participate in viral transcription, DNA replication, and genome packaging, our study further expands its functional role in immune regulation. This work provides new insights into the dual functions of key ASFV structural proteins in viral replication and host immune modulation, and offers important theoretical support for elucidating ASFV-host interactions and identifying potential targets for attenuated vaccine development.

## Ethics declaration

The author(s) declare(s) that the biosafety and ethical approval information for all live ASFV and animal experiments is fully described in Section 2.1 (Biosafety and ethics statement) of the manuscript

## Funding

This research was financially supported by the National Natural Science Foundation of China (Grant No. U24A20450), the Natural Science Foundation of Gansu Province (Grant Nos. 25JRRA436; 24JRRA011; 26JRRA385), the Science and Technology Major Project of Gansu Province (Grant No. 22ZD6NA001), the Science Fund for Creative Research Groups (22JR5RA024) and Special Project (22CX8NA011) of Gansu Province, the Central Public-interest Scientific Institution Basal Research Fund (Grant No. CAAS-ZDRW202409) and the Innovation Program of the Chinese Academy of Agricultural Sciences (CAAS-ASTIP-2021-LVRI).

## CRediT authorship contribution statement

**Xinting Lan:** Writing – original draft, Software, Methodology, Investigation. **Yikang Chen:** Writing – original draft, Software, Methodology, Investigation. **Zhonghui Zhang:** Validation, Methodology, Formal analysis. **Jifei Yang:** Validation, Methodology, Formal analysis. **Rongzeng Hao:** Methodology, Investigation. **Xuesai Li:** Methodology, Investigation. **Hualin Sun:** Methodology, Conceptualization. **Yuting Liao:** Methodology, Conceptualization. **Zhancheng Tian:** Writing – review & editing, Investigation. **Guiquan Guan:** Supervision. **Qingli Niu:** Writing – review & editing, Writing – original draft, Investigation, Funding acquisition, Conceptualization. **Hong Yin:** Writing – review & editing, Writing – original draft, Supervision, Software, Methodology, Investigation, Funding acquisition, Data curation, Conceptualization.

## Declaration of competing interest

The authors declare no competing interests

## Data Availability

Data will be made available on request.
