# Supplementary material for: Transcriptomic analysis reveals enhanced host inflammatory responses associated with TNF-α/NF-κB signaling pathway during infection with the ASFV CN/SC/2019 pA104R-K5R
Source: Virus Res. 2026 Oct 25;373:199822. doi: 10.1016/j.virusres.2026.199822 (PMC13755455; doi:10.1016/j.virusres.2026.199822)
Supplement: Supplementary file 1 [file mmc1.docx]

**Fig. S1. Bar gradients of GO biological process (BP) terms related to immunity and inflammation among different groups.** Analysis of BP terms in WT vs. NC, K5R vs. NC, and K5R vs. WT at 24 hpi. Bar charts show the number of DEGs enriched in each immunity- and inflammation-related term. The color gradient represents the significance level (−log10 Q-value).
